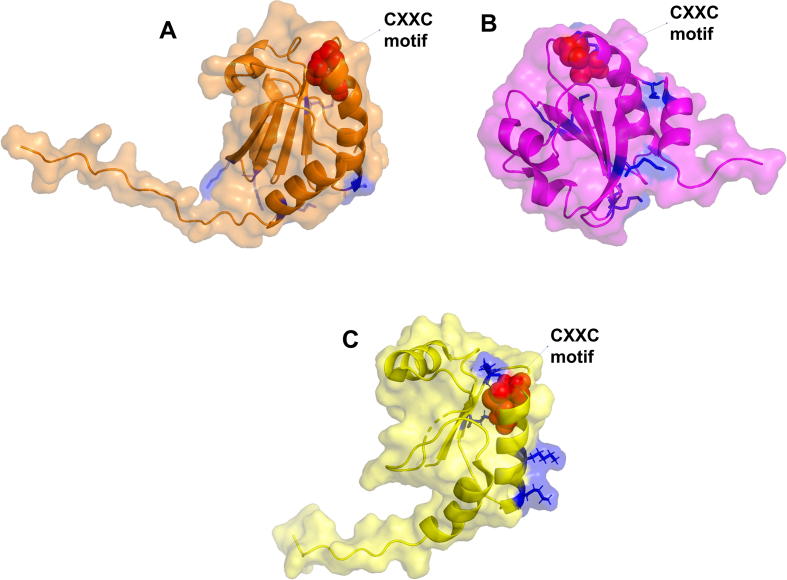# The role of thioredoxin proteins in *Mycobacterium tuberculosis* probed by proteome-wide target profiling

**DOI:** 10.1016/j.bbrep.2023.101512

**Published:** 2023-07-14

**Authors:** Sapna Sugandhi, Vyankatesh Rajmane, Khushman Taunk, Sushama Jadhav, Vijay Nema, Srikanth Rapole, Shekhar C. Mande

**Affiliations:** a National Centre for Cell Science, Savitribai Phule Pune University Campus, Pune, India; b Division of Molecular Biology, ICMR-National AIDS Research Institute, Pune, India

**Keywords:** Thioredoxin, Thioredoxin trapping chromatography, Thioredoxin target profiling, Fe–S cluster biogenesis, Mass spectrometry

## Abstract

*Mycobacterium tuberculosis* encounters diverse microenvironments, including oxidative assault (ROS and RNS), when it attempts to establish itself within its human host. Therefore, redox sensory and regulation processes are assumed significant importance, as these are essential processes for *M. tuberculosis* to survive under these hostile conditions. *M. tuberculosis* contains thioredoxin system to maintain redox homeostasis, which establish a balance between the thiol/dithiol couple.

Still very less is known about it. In the present study, we attempted to capture the targets of all the *M. tuberculosis* thioredoxin proteins (*viz*., TrxB and TrxC) and a thioredoxin-like protein, NrdH, under aerobic and hypoxic conditions by performing thioredoxin trapping chromatography followed by mass spectrometry. We found that TrxC captured the maximum number of targets in both the physiological conditions and most of the targets of TrxB and NrdH showing overlap with targets of TrxC, indicating that TrxC acts as main thioredoxin. Further the PANTHER classification system provides involvement of targets in various metabolic processes and Gene Ontology analysis suggests that glutamine biosynthetic process and Fe–S cluster biosynthesis are the most enriched processes in the target list of TrxC and TrxB respectively. Also, we suggest that the thioredoxin system might play an important role under hypoxia by targeting those proteins which are responsible to sense and maintain hypoxic conditions. Furthermore, our studies establish a link between TrxB and iron-sulfur cluster biogenesis in *M. tuberculosis*. Ultimately, these findings open a new direction to target the thioredoxin system for screening new anti-mycobacterial drug targets.

## Highlights

• Thioredoxin (Trx) system performs a major role to survive under redox stress, generated by the host.
• TrxC captured the highest number of targets under aerobic and hypoxic conditions.
• TrxC acts as a major reducing agent and TrxB is dedicated to perform special functions.
• TrxB and TrxC interact with proteins responsible for survival under hypoxic conditions.
• TrxB performs a specialized function in the Fe–S cluster biogenesis by interacting with Rv1465.

## Graphical abstract

**Figure:**
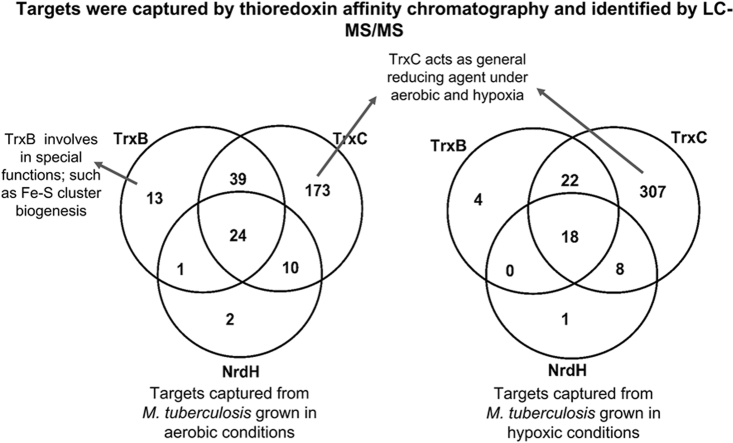

## 1 Introduction

The thioredoxin system of *M. tuberculosis* is composed of one thioredoxin reductase (TrxR) and three thioredoxins designated as thioredoxin A (TrxA), thioredoxin B (TrxB) and thioredoxin C (TrxC), from these, only TrxB and TrxC are capable of accepting electrons from TrxR [1]. Apart from these three genes, the genome of *M. tuberculosis* shows presence of a few thioredoxin-like genes, including *nrdH* which might play a specialized role during oxidation/reduction processes [2,3]. The thioredoxin system of *M. tuberculosis* is mainly responsible for mitigating the undesirable effects of reactive oxygen species, reactive nitrogen species, free radicals etc. Also the genetic inactivation of *M. tuberculosis* TrxR perturbed several growth essential processes with a major effect on susceptibility to thiol-disulfide stress [4]. This indicates the involvement of thioredoxins in various growth essential processes by regulating thiol/disulfide balance. Although, very little is known about the multiple functions of thioredoxin system in *M. tuberculosis*. The first step in understanding these aspects would be to identify partners of thioredoxins and thioredoxin-like proteins under oxygen-rich and oxygen-poor conditions. Since *M. tuberculosis* is known to be subjected to varying levels of O_2_ concentrations during pathogenesis, identification of partners and thereby the redox regulation by thioredoxins enhances our understanding of the response of *M. tuberculosis* to such perturbations through this work. Also, experimental observation of upregulation of thioredoxins in hypoxic conditions in *M. tuberculosis* is suggestive of its role in countering the hypoxic environments of the host [5].

Considering the quintessence of the thioredoxin system under aerobic and hypoxic conditions we aimed to identify the targets of TrxB and TrxC under aerobic and hypoxic conditions. NrdH was also selected as a representative example for identification of its targets under both the conditions because of the presence of thioredoxins like activity in it. We performed the well-established thioredoxin trapping/affinity chromatographic studies [6] to gain further insight into the interactome of *M. tuberculosis* TrxB, TrxC and NrdH. Further to perform the validation studies mycobacterial fragment complementation assay was done.

As a result, we found that TrxC captured the highest number of targets among the three thioredoxins under both the physiological conditions and most of the targets of TrxB and NrdH showing overlap with targets of TrxC, indicating that TrxC acts as main thioredoxin. Our data indicates the versatility of thioredoxins of *M. tuberculosis* under aerobic and hypoxic conditions as these are able to capture important enzymes of metabolic processes. To the best of our knowledge this is the first report on *M. tuberculosis* in suggesting the possible involvement of thioredoxins in major metabolic processes. Also, we suggest that the thioredoxin system possibly play an important role under hypoxia by targeting those proteins which are responsible to sense and maintain hypoxic conditions. Further, our study also suggests a role of TrxB in Fe–S cluster biogenesis by showing interaction of TrxB and Rv1465 in *M. tuberculosis.*

## 2 Materials and methods

### 2.1 Cloning and purification

*M. tuberculosis trxB* (Rv1471), *trxC* (Rv3914) and *nrdH* (Rv3053c) were previously cloned in our lab using pET23a, pET22b and pET21a expression vectors respectively [1,2]. Single point mutants of *trxB*_C33S, *trxC*_C40S and *nrdH*_C14S were created by site directed mutagenesis. Primers were designed to incorporate the mutations in the gene by PCR amplification (Supplementary Table 1). The parental strand was cleaved using *Dpn*I and further *E. coli* TOP10 strain was transformed with the digested products. Colonies obtained were subjected to isolate plasmids and these plasmids were sequenced to confirm the incorporation of point mutant.

Plasmids having *trxB*_C33S, *trxC*_C40S and *nrdH*_C14S were transformed into *E. coli* BL21 (DE3) strain to purify protein. Briefly, the transformants were cultured at 37°C in Luria broth (LB-Himedia) supplemented with 100 μg/ml ampicillin. Protein expression was induced with the addition of 0.5 mM IPTG for 4 h at 37°C when O.D. at 600 nm reached 0.6. After cell lysis, the lysate was subjected to high-speed centrifugation to remove cellular debris. The supernatant was allowed to bind with nickel-nitrilotriacetic acid (Ni-NTA) resin, pre-equilibrated with a buffer containing 50 mM Tris-Cl at pH 8, 300 mM NaCl, 5% glycerol, and 10 mM imidazole. This resin was washed with a buffer containing 50 mM Tris-Cl at pH 8, 300 mM NaCl, 5% glycerol, and 20 mM imidazole to remove contaminants. The final elution was done with a buffer containing 50 mM Tris-Cl pH 8, 300 mM NaCl, 5% glycerol, and 250 mM imidazole. Fractions containing protein were collected and subjected to size exclusion chromatography. HiLoad 16/600 Superdex 75 (Cytiva life sciences) gel filtration column was used for the further purification of TrxB C33S, TrxC C40S and NrdH C14S with buffer containing, 10 mM Tris-Cl pH 8, 150 mM NaCl. The elution fractions were analyzed on SDS-PAGE to detect the presence of purified protein.

### 2.2 *M. tuberculosis* culture

*M. tuberculosis* H37Rv strain was cultured in 7H9 medium (Himedia), supplemented with 10% (v/v) ADC (bovine albumin fraction V, dextrose, catalase). For aerobic conditions, culture was grown at 37°C in shaking conditions until O.D. at 600 nm reached 0.6–0.8. For hypoxia optimized protocol from the studies of Sharma et al. was opted [7]. Briefly, culture with 0.6–0.8 O.D. at 600 nm was kept standing in a tightly sealed 50 ml centrifuge tube with no head space left for 7 days at 37°C Both the cultures were harvested at 4000 rpm for 10 min, and then the pellet was resuspended in 1 ml of lysis buffer containing 1X phosphate buffer saline pH 7.6 with 1X protease inhibitor cocktail, after washing with 1X phosphate buffer saline. Cells were lysed by bead beating method for 10 times with 20 second pulse on and 2 min pulse off on the ice. Supernatant was collected and filtered with a 0.2 μm filter, after centrifugation at 13,000 rpm for 15 min.

### 2.3 Pull down assay

Targets of TrxB, TrxC and NrdH were captured by well-established thioredoxin affinity chromatography [6] Targets were captured by immobilized TrxB C33S, TrxC C40S and NrdH C14S, mutant proteins to the cyanogen bromide (CNBr) activated sepharose 4 b resin (Merck). Resin was activated by swelling it into 1 mM HCl according to the manufacturer's instructions and equilibrated with a coupling buffer (20 mM sodium phosphate pH 8, 300 mM NaCl). A total of 1 mg of each protein was immobilized on 50 μl of resin and incubated overnight at 4°C. Unbound protein was washed with the coupling buffer. After binding of protein to the resin, blocking was done at room temperature for 2 h with 100 mM Tris-Cl pH 8 to block unmodified unreactive groups of resin. *M. tuberculosis* cell lysate containing 6–8 mg protein was incubated with 50 μl of protein bound resin for 2 h at room temperature under gentle stirring conditions. Further washing with 10 mM Tris-Cl pH 8, 300 mM NaCl was done to remove nonspecific interactions. Washing step was performed until O.D. at 280 nm of the washing solution was reached to zero. Finally, for elution 60 μl of 10 mM Tris-Cl pH 8 containing 10 mM dithiothreitol (DTT) was incubated with 50 μl of resin for 1 h at room temperature. Eluted proteins were subjected to liquid chromatography-tandem mass spectrometry analysis to identify the targets.

Activated and equilibrated resin without the protein was used and incubated with *M. tuberculosis* cell lysate as a control experiment. All the experimental conditions were kept constant for the control experiment.

### 2.4 In-solution digestion and LC-MS/MS proteomic analysis

Solution based protein digestion was performed as per the protocol described previously using samples containing targets of thioredoxin [8]. Briefly, each proteome sample was reduced with 10 mM DTT, alkylated with 50 mM iodoacetamide and subjected to protein digestion using the proteolytic enzyme trypsin (1:50; enzyme:protein). Furthermore, the digested tryptic peptides from the pull-down samples and from the control group were desalted using C18 ziptips (EMD Millipore). Samples were further reconstituted in liquid chromatography-mass spectrometry (LC-MS) grade water (J.T.Baker; Thermo Fisher Scientific, Inc.) containing 0.1% v/v formic acid (Sigma Aldrich; Merck KGaA).

Orbitrap Fusion™ mass spectrometer (Thermo Fisher Scientific, Inc.) coupled to an EASY-nLC™ 1200 nano-flow LC system (Thermo Fisher Scientific, Inc.) equipped with EASY-Spray column (50 cm × 75 μm ID; PepMap C18 column) was used for data acquisition. For each MS data acquisition, 1 μg desalted tryptic peptides from each sample were injected into the Orbitrap Fusion mass spectrometer. Peptides were separated using a 5%–95% solvent B, containing 0.1% V/V formic acid in 80% V/V LC-MS grade acetonitrile at a flow rate of 300 nL/min for 140 min gradient process. The solvent consisted of 0.1% V/V formic acid in LC-MS grade water. The mass spectra were acquired in positive ionization mode with positive ionization spray voltage of 2 KV. The MS scan began with an analysis of MS1 spectrum from the mass range 375–1500 *m/z*. Analysis was performed using the Orbitrap analyzer at a resolution of 60,000; with an automatic gain control (AGC) target of 4 × 10^5^ and maximum injection time of 50 msec. The precursors identified in MS1 were fragmented by high energy collision-induced dissociation and analyzed using the Orbitrap mass analyzer (NCE 35; AGC 1 × 10^5^; maximum injection time 40 msec, resolution 15,000 at 200 *m/z*).

The MS data was analyzed to identify proteins using the Proteome Discoverer software (version 2.2; Thermo Fisher Scientific, Inc.). This was carried out by employing the Sequest HT database search engine with 1% false discovery rate (FDR) and the cut-off criteria of 2 missed cleavages. Database searching included all entries from the *M. tuberculosis* proteome database, UniProt reference proteome number UP000001584 and download date September 02, 2020. Total protein level analysis was performed using a 10 parts per million precursor ion tolerance. The product ion tolerance used for the data analysis was 0.05 Da. Oxidation of methionine residues (+15.995 Da) was kept as a variable modification, whereas carbamidomethylation of cysteine residues (+57.021 Da) was kept as a static modification. Peptide-spectra matches (PSMs) were adjusted to an FDR of 0.01. PSMs were identified, quantified (using MS/MS fragment intensity), and narrowed down to a 1% peptide FDR and then further narrowed down to a final FDR protein level of 1% for protein-level comparisons. The sum of the area of peptide ions across all matching PSMs was used for protein quantification. The criteria to accept a protein identity was, presence of minimum 3 unique peptide and the coverage should be equal to/greater than 30%.

### 2.5 Data analysis

All experiments were done in duplicate to identify the targets of thioredoxins proteins under both aerobic and hypoxic conditions. Common proteins from both the duplicate sets were further studied. Thioredoxins majorly act on substrates which possess cysteine residues and therefore only cysteine-containing proteins were screened out from the total proteins captured. The overlap of targets was also checked between the TrxB, TrxC and NrdH.

For further analysis, the classification of targets was done into biological processes using the PANTHER classification system. The PANTHER classification system allows the study of complex proteomics data by combining gene function, ontology, pathways and statistical analysis tools [9].

The GO online tool (http://www.geneontology.org/) was used for enrichment analysis of targets of thioredoxins [10]. UniProt identifier of targets were entered into the tool, where *M. tuberculosis* was selected as an organism and biological process was selected for ontology analysis. Processes with highest fold enrichment value and p-value <0.05 were selected further.

In order to understand the significance of thioredoxins under hypoxia, list of upregulated *M. tuberculosis* genes under hypoxia was retrieved from existing literature. This list was compared with the targets of thioredoxins captured from cell lysate of *M. tuberculosis* grown under hypoxic conditions to understand the significance of thioredoxins in entering, exiting or enduring hypoxic response. Data used from Gene expression omnibus, reported by the upregulation studies of Matern et al. [11,12].

### 2.6 Mycobacterial protein fragment complementation assay

M-PFC assay was done on the basis of the previously described method [13]. Vectors, pUAB300(F1,2), pUAB400(F3), pUAB100-GCN4[F1,2] and pUAB200-GCN4[F3] are available to check interaction between mycobacterial proteins. Constructs pUAB300-*trxB*(F1,2), and pUAB400-*Rv1465*(F3) were made by restriction enzyme cloning approach. Electrocompetent cells of *M. smegmatis* were transformed with combination of pUAB300-*trxB*(F1,2)/pUAB400-*Rv1465*(F3) by electroporation and allowed to grow on 7H10 agar (Himedia) supplementing with 10% (v/v) OADC (Oleic Albumin Dextrose Catalase), hygromycin (50 μg/ml) and kanamycin (25 μg/ml). Transformants were further cultured on 7H11 agar supplementing with 10% (v/v) OADC, hygromycin, kanamycin and trimethoprim to check the protein-protein interaction. A combination of pUAB100-GCN4[F1,2]/pUAB200-GCN4[F3] was used as a positive control in the M-PFC assay, whereas pUAB200(F3)/pUAB300(F1,2) was used as a negative control.

### 2.7 Fe–S cluster reconstitution on TrxB

Chemical mode of the Fe–S cluster reconstitution was applied to incorporate Fe–S cluster on TrxB, as described by Freibert et al. with minor modifications [14]. All stocks were freshly prepared and degassed. Briefly, FeCl_3_ as an iron source and Li_2_S as a source for sulfur were used to incorporate the Fe–S cluster on TrxB. A total of 1 mM TrxB was reduced with 5 mM DTT at room temperature for 1 h in anaerobic chamber. For the subsequent Fe–S cluster reconstitution, TrxB was diluted to the final concentration of 150 μM with a buffer containing 50 mM Tris-Cl pH 8, 150 mM NaCl and 2 mM DTT. FeCl_3_ was added to the final concentration of 600 μM and incubated for 5 min at room temperature. Li_2_S was added at the same molar concentration as FeCl_3_ and incubated for 10 min at room temperature under anaerobic chamber. Further to remove unbound reaction components, the reconstituted reaction was allowed to bind with 100 μl of Ni-NTA resin, pre-equilibrated with 50 mM Tris-Cl pH 8, 150 mM NaCl for 10 min. Washing was done for 10 column volumes with a buffer containing 50 mM Tris-Cl pH 8, 150 mM NaCl. Reconstituted TrxB was eluted with a buffer containing 50 mM Tris-Cl pH 8, 150 mM NaCl, 250 mM imidazole. To confirm the Fe–S cluster reconstitution, absorbance at 420 nm was observed.

## 3 Results and discussion

Thioredoxins being the major buffer against redox fluctuations in cells, it is important to understand the mechanism of how they regulate response to redox changes. Typically, thioredoxins participate in these processes by thiol disulfide exchange reactions and by partnering with different proteins in the cells, protecting them from such perturbations. Thioredoxins have the ability to attack on the substrates having S–S bridge and reduce them. Thioredoxins contain active site CXXC motif. One Cys with lower pKa value (attacking Cysteine) acts as a nucleophile (thiolate ion). The thiolate ion attacks on the disulfide bond of the target and forms a mixed disulfide bond between target and enzyme. At this stage another Cys residue (resolving Cysteine) attacks on the mixed disulfide bond, leaving the target in reduced form.

**Fig. 1:**
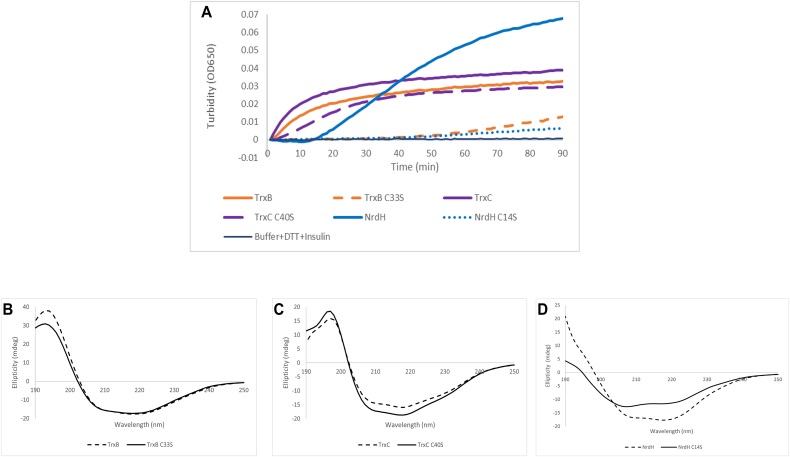
Insulin precipitation assay and Far-UV CD spectra. (A) Insulin turbidity was measured at 650 nm (on Y-axis) and plotted as a function of time in minutes (on X-axis). (B), (C), (D) Far-UV CD spectra of wild type (solid line) and mutant (dash line) of (B) TrxB, (C) TrxC and (D) NrdH. Y-axis represents ellipticity (mdeg) and X-axis represents wavelength (nm).

*M. tuberculosis* has been shown to possess three thioredoxins, out of which two are known to be active, and some glutaredoxin-like proteins, such as NrdH with thioredoxin-like activity [1,2]. Thioredoxin affinity chromatography technique was used to capture the targets, which uses CNBr activated Sepharose resin, that interacts with primary amine group. TrxB, TrxC and NrdH contains 5, 9 and 6 number of Lys residues respectively, which are able to interact with CNBr resin and immobilized the protein. For TrxB all Lys residues are positioned away from active site and therefore active site is free to interact with targets. For TrxC and NrdH, only one Lys residue is positioned near to active site (Supplementary Fig. 1). Except this one condition of TrxC and NrdH, all other positions with free active site are available to interact with resin and capture targets. Resolving cysteine mutants were used to capture the targets of these thioredoxins. All the resolving cysteine mutants retain reduction activity. Thioredoxins displays the ability to reduce disulfide bond present in insulin and leads to the precipitation of its β-chain. This property was used to check the activity of *M. tuberculosis* thioredoxins; TrxB, TrxC NrdH and its mutants; TrxB C33S, TrxC C40S, NrdH C14S. All the proteins were able to reduce disulfide bond of insulin. Although, the activity was reduced in mutants as compared to wild type proteins. These mutants were able to reduce the disulfide bond formed between the thioredoxin and target but unable to resolve it (Fig. 1A). Far-UV circular dichroism experiments confirmed that, there are no significant changes in secondary structure of mutant proteins of TrxB and TrxC as compared to wild type proteins. (Fig. 1B–C). Overall secondary structure remains the same for mutants of TrxB and TrxC. This suggests that Cys to Ser mutation do not affect the folding of the thioredoxins. For mutant of NrdH there is minor change in secondary structure as compared to the wild type (Fig. 1D).

In the present study we identified the proteome-wide targets of two main thioredoxins, TrxB and TrxC in aerobic and hypoxic culture conditions. We also selected NrdH to identify targets as representative example of thioredoxin-like protein. NrdH has not been identified as the principle redox buffer for *M. tuberculosis*. However, its thioredoxin-like activity and ability to accept electrons from TrxR encouraged us to provide insights into the exclusive role of thioredoxin-like proteins in *M. tuberculosis*. Since *M. tuberculosis* is known to be subjected to varying levels of O_2_ concentrations during pathogenesis, identification of partners and thereby the redox regulation by thioredoxins enhances our understanding of the response of *M. tuberculosis* to such perturbations through this work.

### 3.1 Target identification of thioredoxins from *M. tuberculosis*

TrxB was able to capture 77 Cys-containing proteins from *M. tuberculosis* cell lysate grown under aerobic conditions and 44 proteins from lysate grown under hypoxic conditions. Whereas, TrxC captured 246 Cys-containing proteins from cell lysate grown under aerobic conditions and 355 proteins from lysate grown under hypoxic conditions. Similarly, NrdH was able to capture 37 Cys-containing proteins from lysate prepared under aerobic conditions and 27 proteins from *M. tuberculosis* cell lysate prepared under hypoxic conditions (Supplementary Table 2). (List of non-Cys containing proteins is provided in Supplementary Table 3.) These Cys-containing proteins were considered as possible targets for thioredoxins in *M. tuberculosis*.

The total number of proteins captured from both the growth conditions clearly indicates that under aerobic conditions there is an increased number of targets of TrxB when compared with hypoxic conditions, suggesting that TrxB is likely to be more active under aerobic conditions. Whereas, increased number of targets of TrxC from hypoxic conditions suggest the involvement of TrxC under hypoxia. Overall, among all the three proteins, TrxC captured the maximum number of targets from both the physiological conditions, whereas TrxB and NrdH captured a much smaller number of targets compared to targets of TrxC.

**Fig. 2:**
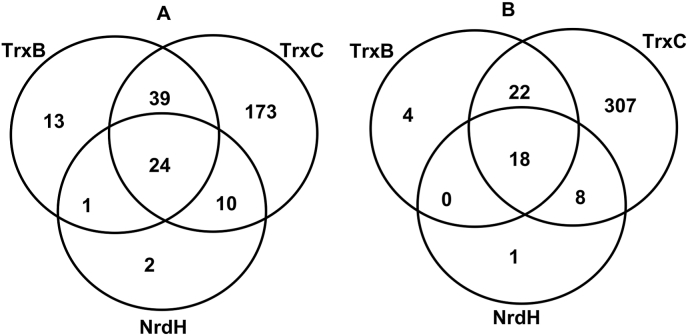
Overlap of targets captured by TrxB, TrxC and NrdH under aerobic conditions (A), and under hypoxia (B).

Moreover, to understand the specificity of thioredoxins towards the targets, targets of TrxB, TrxC and NrdH were overlapped for both the physiological conditions. Analysis of overlap also shows that most of the targets of TrxB and NrdH are shared with TrxC (Fig. 2A–B). Interestingly, NrdH shares 25 common proteins with the target list of TrxB and 35 proteins with targets of TrxC with very few unique targets of its own. The overlap between targets of thioredoxins was clearly indicates the redundancy in the thioredoxin system, possibly offering additional protection to *M. tuberculosis* against redox imbalance. Thus, a large number of targets of TrxC from both the growth conditions, and the significant overlap of TrxB and NrdH targets with those of TrxC, suggests that TrxC might be the principal thioredoxin in *M. tuberculosis*.

Top 3 most abundant proteins were further identified from the targets captured by TrxB, TrxC and NrdH. For TrxB protein, thiol peroxidase (TpX), Rv2204c and Rv0250c are the most abundant targets from aerobic conditions and from hypoxia, IdeR, Putative thiosulfate sulfurtransferase (CysA1) and Rv3489 are the most abundant. Similarly, the most abundant protein from the target captured by TrxC from aerobic conditions are; CysA1, Probable fatty acid synthase (Fas), Glycerol-3-phosphate dehydrogenase (GpdA2) and from hypoxic conditions are; Rv0887. For NrdH protein; CysA1, TpX, IdeR are the most abundant targets from aerobic conditions and from hypoxia, CysA1, TpX and Rv3489 are the most abundant. Most of the top abundant targets are common for TrxB, TrxC and NrdH and they are involved in the active transport across the membrane of multiple sulfur-containing compounds with the help of CysA1 and in antioxidant activity using TpX. Moreover, targets of TrxC are involved in fatty acid and carbohydrate metabolism with Fas and GpdA2 enzymes.

The presence of a maximum number of common targets of NrdH with TrxB and TrxC, suggest that although NrdH might not be the principal reductant, yet it offers redundancy to the system in sharing targets with other thioredoxins. The role of thioredoxin-like proteins in *M. tuberculosis* therefore might be that of offering redundancy to redox response to the cells, or being specialized reductants of a selected few processes. But in the present study no such processes come out which are specific for NrdH.

### 3.2 Significance of thioredoxins under hypoxia

We found that thioredoxins interact with many proteins which are upregulated under hypoxic conditions. Comparing the present data with upregulation studies, a total of 21 targets of TrxB and 124 targets of TrxC were seen to be upregulated under hypoxia [12]. A high number of targets of TrxC under hypoxia and common targets with TrxB, indicates that TrxC might be the main thioredoxin in hypoxic conditions also.

Hypoxia acts as a trigger for *M. tuberculosis* to switch its metabolic processes, and thereby survival under hypoxic conditions is an important aspect of *M. tuberculosis* physiology [15]. In spite of being an obligate aerobe, it shifts the entire metabolism to anaerobic mode of respiration. *M. tuberculosis* follows glyoxylate cycle when tricarboxylic acid cycle is down regulated upon oxygen depletion [16]. Isocitrate lyase is the key enzyme of glyoxylate cycle, which reversibly cleaves the isocitrate to glyoxylate and succinate. In our study, TrxC was found to interact with isocitrate lyase. Switching carbon source from carbohydrates to lipid is another route to provide energy during hypoxia. FadA6, Fad5 and Fad15 are involved in lipid degradation to facilitate the use of lipid carbon source in *M. tuberculosis.* These were also found to interact with TrxC in our study, suggesting the involvement of thioredoxin in carbon metabolism under hypoxic conditions.

In the present study, the enzymes that are required to sense and execute responses against hypoxia are also found to be targeted by thioredoxins. For example, TrxB and TrxC were showing interaction with Rv2624c, which is a hypoxic response protein, and with Rv2646c and Rv2623, which are universal stress proteins in *M. tuberculosis*. DosR which is a key component of the DosR/DosS/DosT regulon also interacts with TrxC under hypoxic conditions thus underscoring the role of thioredoxin in the survival of the organism in hypoxic conditions. Interaction between TrxC and DosR might be the master regulator of redox dependent interactions and activation of hypoxia-related transcription factors by thioredoxins in *M. tuberculosis*. Thioredoxins in many other species are known to facilitate activation/inactivation of transcription factors and subsequently trigger transcriptional response. For example, human thioredoxin is involved in redox regulation of various transcription factor such as, NFκB [17], p53 [18] and Sp1 [19]. The interaction of thioredoxins with hypoxia responsive/regulatory proteins therefore suggests a role of thioredoxin in survival of the *M. tuberculosis* in hypoxic conditions.

### 3.3 Protein annotation through evolutionary relationship (PANTHER) classification and gene ontology (GO) analysis

**Fig. 3:**
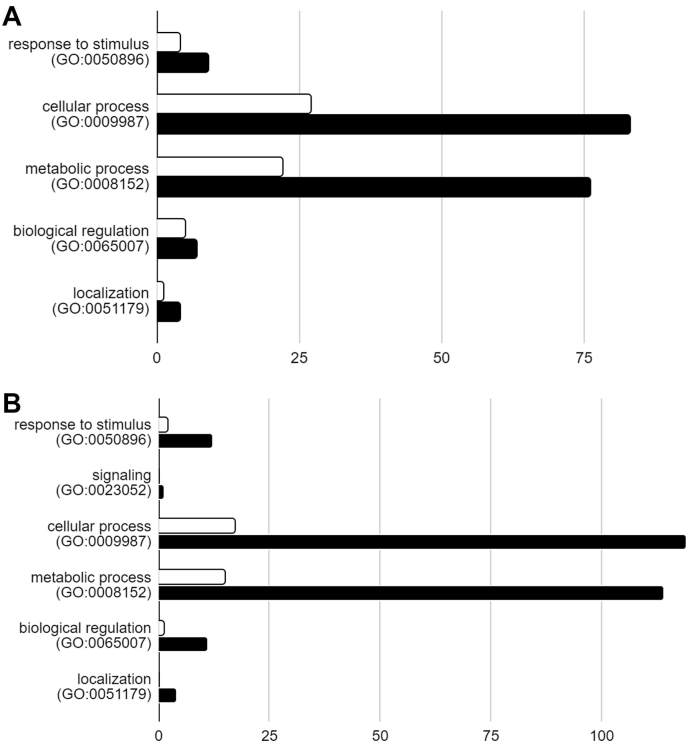
Classification of targets captured by TrxB and TrxC, under aerobic (A) and hypoxic (B) conditions. Classification was done into biological processes using PANTHER classification system. Y-axis showing biological processes and X-axis showing number of proteins involved in that processes. White bars represent targets of TrxB and black bars represents targets of TrxC.

**Fig. 4:**
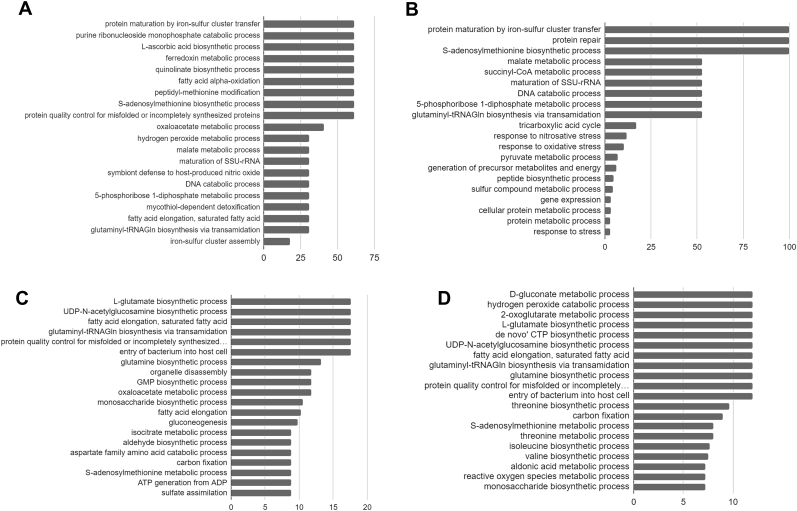
Gene ontology analysis of targets captured by TrxB under aerobic condition (A), TrxB under hypoxic condition (B), TrxC under aerobic condition (C) and TrxC under hypoxic condition (D). X-axis represents fold enrichment value and Y-axis represents name of enriched processes. Bar chart showing the top 20 GO terms for biological process ranked by fold enrichment of all targets.

PANTHER classification system classified the targets into different processes which include; metabolic processes, cellular processes, biological regulation, response to stimulus, localization and signaling process (Fig. 3A–B). For TrxB and TrxC, most of the targets were involved in cellular processes and metabolic processes in both the physiological conditions. GO enrichment analysis of targets reveals the most enriched processes among all by providing fold enrichment values. For TrxB and TrxC the top 20 processes ranked based on fold enrichment score are shown in Fig. 4 (A-D). From these classes, some processes are highly enriched and therefore further described below.

Further, GO analysis of targets of TrxC captured from both the conditions suggest that TrxC is able to capture targets which are involved in metabolic pathways of amino acid, carbohydrate, fatty acid (Fig. 4C–D). The above observations and findings suggest that TrxC might be a generalized reducing agent for *M. tuberculosis.*

### 3.4 Target analysis of TrxC

The glutamate biosynthetic process and the fatty acid elongation process have highest fold enrichment values from the targets of TrxC captured from lysate prepared under aerobic conditions and carbon fixation and glutamine biosynthetic processes have highest values of fold enrichment from hypoxia. Glutamine synthetase (Gln) and glutamate synthase (GOGAT-large subunit GltB and small subunit GltD) together perform ammonia assimilation in *M. tuberculosis*. In *M. tuberculosis* Gln is involved in cell wall synthesis, and in coping with nitrosative and acidic stress. In the present study glutamate synthase and glutamine synthetases are found to interact only with TrxC, which indicates that in *M. tuberculosis* this function is mainly governed by TrxC. Interaction of thioredoxin protein with GS-GOGAT has been previously shown in *Chlorella pyrenoidosa, Chlorella sorokiniana*, *Anabaena cylindrica. In Vitro* studies on GS-GOGAT and thioredoxin from *Chlorella pyrenoidosa, Chlorella sorokiniana*, *Anabaena cylindrical* showed enhancement of GS-GOGAT activity in presence of thioredoxins and strong reducing agent dithioerythritol [[20], [21], [22]]. Therefore, we hypothesize that redox regulation of nitrogen metabolism in *M. tuberculosis* is similarly governed by TrxC because of its interaction with glutamine synthetase and glutamate synthase.

Another notable finding of our study is the interaction of NrdE and NrdF1 (ribonucleotide reductase) with TrxC. Reduction of ribonucleotide reductase by thioredoxin proteins is well studied in all forms of life. In *M. tuberculosis* NrdH has been proposed to act as a reducing agent for ribonucleotide reductase, but no experimental evidence is available [2,23]. Interestingly, our data shows no interaction of NrdE with NrdH. However, in this data TrxC is found to be interacting with NrdE and NrdF1. TrxB also shows interaction with NrdF2. NrdF is known to generate free radicals that are required for catalysis and NrdE serves as an electron acceptor from reducing agents such as the thioredoxin system. The active physiological complex of NrdE and NrdF allows the possibility of targeting NrdF by thioredoxins from the findings of the present study.

### 3.5 Target analysis of TrxB

From targets captured by TrxB, the Fe–S cluster transfer process and purine ribonucleoside monophosphate catabolic process from aerobic conditions and again Fe–S cluster transfer process by Rv2204c and S-adenosylmethionine biosynthetic process from hypoxic conditions are present on top position in GO enrichment value. Presence of processes related to Fe–S cluster biogenesis in GO analysis of targets of TrxB captured from aerobic conditions indicate that the TrxB is involved in the Fe–S cluster related processes. These processes are; proteins maturation by Fe–S cluster transfer, ferredoxins metabolism and Fe–S cluster assembly process (Fig. 4A). According to gene ontology analysis Fe–S cluster assembly process contains 7 proteins, out of which Rv2204 and Rv1465 are showing interaction with TrxB. Rv1465 is a NifU like protein transcribed by *suf* operon of *M. tubercolosis.* It is homologous of SufU of *B. subtilis* and essential for *M. tuberculosis* [24]. The role of Rv1465 is to accept the persulfide moiety from Rv1464 (cysteine desulfurase) on its surface and then transfer it to scaffold proteins for the synthesis of Fe–S cluster.

### 3.6 Interaction of TrxB and Rv1465

**Fig. 5:**
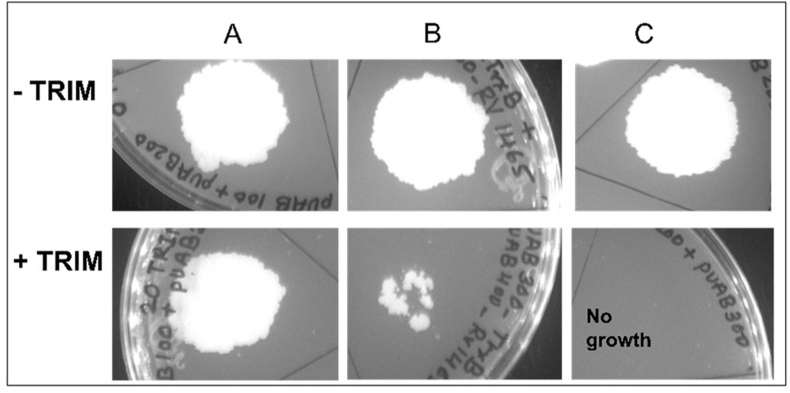
M-PFC assay for detecting the interaction between TrxB and Rv1465 in *M. smegmatis*. *M. smegmatis* cells were co-transformed with plasmids and transformants were subculture on 7H11 media (having 25 μg/ml kanamycin and 50 μg/ml hygromycin) with or without Trimethoprim. (A) pUAB100-GCN4[F1,2]/pUAB200-GCN4[F3] as positive control. (B) pUAB300-*trxB*(F1,2)/pUAB400-*Rv1465*(F3). (C) pUAB300-*RV1465*(F1,2)/pUAB400-*trxB*(F3). (D) pUAB200(F3)/pUAB300(F1,2) – negative control.

Considering the essentiality of Rv1465 for *M. tuberculosis* in Fe–S cluster biogenesis, Rv1465 was selected to validate the finding. TrxB captured Rv1465 in aerobic conditions. Target validation was done using M-PFC assay. To perform M-PFC assay, constructs were designed using pUAB300(F1,2) and pUAB400(F3), in such a way that both the fragments of mDHFR (F1,2 and F3) interacted only if interaction between proteins of interest was there. This interaction provides the viability of transformants on the 7H11 medium containing trimethoprim. Therefore, the interaction between TrxB and Rv1465 was confirmed by the presence of viable phenotype on 7H11 media containing 20 μg/ml trimethoprim. Absence of growth on the combination of pUAB200 and pUAB300 represents no interaction between both the fragments of mDHFR. The combination of pUAB100-GCN4[F1,2]/pUAB200-GCN4[F3], provides the viable phenotype on the 7H11 medium containing trimethoprim and acts as positive control for the study. Similarly, the interaction of TrxB and Rv1465 allows the functional reconstitution of both the fragments of mDHFR and displayed viable phenotype of co-transformants containing, *MtbtrxB*(F1,2) in pUAB300 and *MtbRv1465*(F3) in pUAB400 on media containing 20 μg/ml trimethoprim (Fig. 5).

### 3.7 *In-vitro* Fe–S cluster reconstitution on TrxB

Apart from the interaction of TrxB with cluster biosynthesis proteins we also observed the unexpected brown-red color on theNi-NTA affinity purified recombinant fraction of TrxB expressed in *E. coli.* We speculated that the color might be a typical coloration property of Fe–S clusters. This observation was later supported when spectroscopic studies on Ni-NTA affinity purified fraction of TrxB provided a characteristic peak of the Fe–S cluster at 420 nm (Data not shown). Interestingly the coloration property was only observed on TrxB, but not on TrxC. This observation encouraged us to perform i*n vitro* Fe–S cluster reconstitution experiments.

**Fig. 6:**
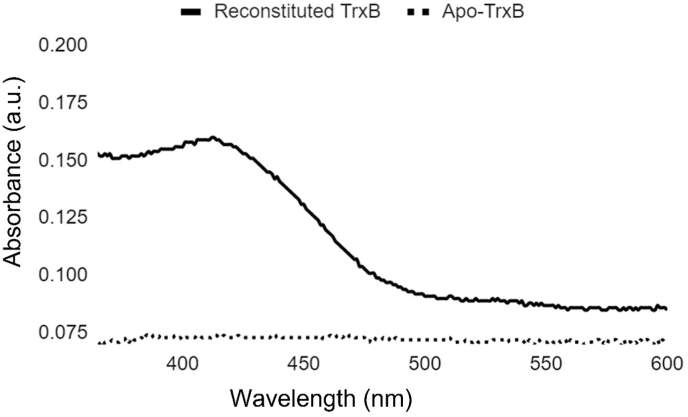
UV–vis spectroscopic characterization of TrxB. Dash line indicates the apo-TrxB and the black line indicates the Fe–S cluster reconstituted TrxB. Both data sets were normalized using the reaction buffer as control. The clear peak at 420 nm specifies the presence of the Fe–S cluster on TrxB.

To validate the presence of Fe–S cluster on TrxB, chemical method of *in vitro* reconstitution of Fe–S cluster was done. As a result, reconstituted reaction showed the presence of characteristic peak at 420 nm indicates the successful reconstitution of Fe–S clusters on TrxB (Fig. 6). Aerobically purified TrxB in identical buffer conditions with reconstituted reaction was selected as control reaction. This control reaction showed no peak at 420 nm, because of the dissociation of cluster during the purification of TrxB in aerobic conditions.

A similar observation to our present study was made in *B. subtilis*, which reported the interaction of SufU (sulfurtransferase) with thioredoxin and presence of Fe–S cluster on thioredoxin [25]. Further, using persulfide reduction assays they showed the enhanced rate of persulfide reduction by SufS-SufU pair in the presence of thioredoxin. Our study is in agreement with the above findings in *B. subtilis* by providing the *in vivo* data of interaction between TrxB and Rv1465 using M-PFC assay. Moreover, result of i*n-vitro* Fe–S cluster reconstitution experiment confirms the binding of Fe–S cluster on TrxB by providing a peak at 420 nm. The binding of the Fe–S cluster to TrxB is an interesting and unexpected finding of our study.

On the basis of evidences of interaction between TrxB and Rv1465 and previously available literature, we hypothesized the role of TrxB in Fe–S cluster biogenesis. The function of Rv1465 is to perform the transfer of terminal sulfide from Rv1464 to scaffold proteins for the synthesis of Fe–S cluster. We hypothesized that this step of terminal sulfide transfer requires a reductant and possibly that reductant is TrxB. Interestingly, we found that Rv1465 was captured exclusively by TrxB Whereas, TrxC captured Rv1461, Rv1462 and Rv1463, which together serve as scaffold for synthesis of Fe–S cluster. Here both the thioredoxins displayed the specificity for their targets despite having similar modes of action. The present study suggests the specific interaction between TrxB with Rv1465 while TrxC acts as a main reductase and interacts with multiple SUF proteins to maintain the redox balance.

**Fig. 7:**
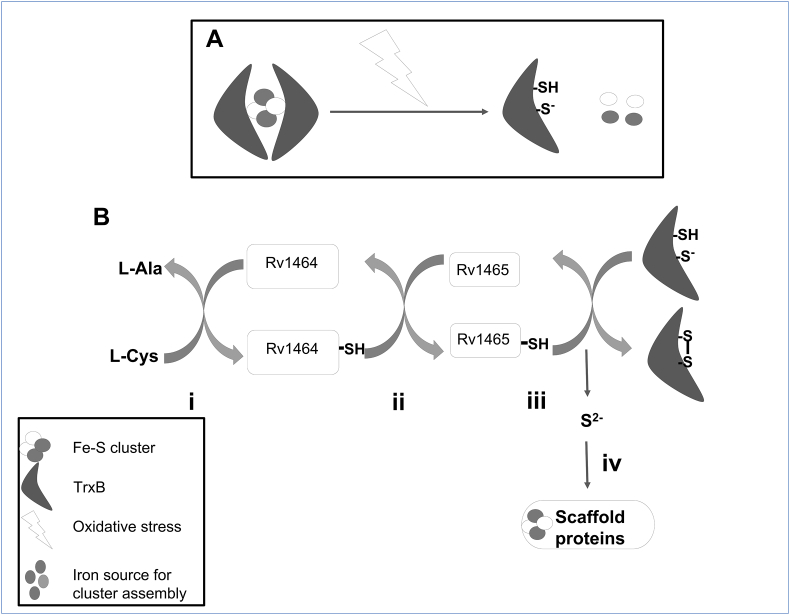
Proposed hypothetical model. A) Proposed hypothesis mode of action of TrxB under oxidative stress condition. Binding of Fe–S clusters causes the dimerization of TrxB and inhibits the reduction activity by blocking the active site CXXC motif. Oxidative stress causes the dissociation of clusters and makes CXXC motif available to perform a reduction function. B) Proposed model for involvement of TrxB in Fe–S cluster biogenesis. i,ii) Cysteine desulfurase reaction mediated by cysteine desulfusare Rv1464, and transfer of the terminal sulfur of the persulfide to Rv1464. iii) Transfer of the terminal sulfur of the persulfide from Rv1464 to its sulfur acceptor protein Rv1465. iv) Reduction of Rv1465 bound persulfide and transfer the terminal sulfur to scaffold protein by TrxB. v) Fe–S cluster synthesis on scaffold protein and transfer to apo proteins.

Overall, we hypothesized that the reduction of persulfide present on Rv1465 and its transfer to scaffold is mediated by TrxB in *M. tuberculosis* (Fig. 7). The binding of TrxC with scaffold proteins could suggests the involvement of TrxC in cluster transfer to apo proteins. We also hypothesized that cluster binding on TrxB could facilitate the oligomerization of TrxB with coordination of catalytic cysteine residues of monomers and iron present in the cluster. This Fe–S cluster binding could be the reason for the loss of reduction function and further activation of enzymes requires accessibility of catalytic cysteine by removal of cluster. Possibly the removal of the cluster could be mediated by oxidative stress encountered by the host. The relevance and relation of Fe–S cluster binding and interaction with cluster biosynthesis protein therefore will need to be further investigated. Overall, this study revealed some novel interactions of thioredoxins such as involvement of thioredoxin in Fe–S cluster biosynthesis. The presence of Fe–S cluster on TrxB opens a new direction to study the role of cluster on thioredoxins. The presence of Fe–S cluster proposes the question of whether TrxB can act as oxidative stress sensor and what would be the effect of cluster binding on reduction property on thioredoxin?

All these findings suggest the role of the thioredoxin system by exploring the new targets in *M. tuberculosis*. Therefore, this study further opens a new direction to discover the novel anti-mycobacterial drugs targeted to the thioredoxin system in future research.

## Author contributions

Contribution of authors is as following: (1) The conception and design of the study: SCM and SS. Acquisition of data: SS, VR, KT, SJ. Analysis and interpretation of data: SS, SCM, SR, VN. (2) Drafting the article and revising it critically for important intellectual content: SS, SCM. (3) Final approval of the version to be submitted: All authors.

## Formatting of funding source

The authors acknowledge the 10.13039/501100001407Department of Biotechnology, Government of India (BT/PR10855/BRB/10/1330/2014) for funding Orbitrap mass spectrometer to NCCS. We also thank the 10.13039/501100001407Department of Biotechnology, Government of India for support through Centre of Excellence grant (BT/PR15450/COE/34/46/2016) to SCM.

## Declaration of competing interest

The authors report no competing interests.

## Figures and Tables

**figs1:**