# Supplementary material for: The role of thioredoxin proteins in Mycobacterium tuberculosis probed by proteome-wide target profiling
Source: Biochem Biophys Rep. 2023 Jul 14;35:101512. doi: 10.1016/j.bbrep.2023.101512 (PMC10371808; doi:10.1016/j.bbrep.2023.101512)
Supplement: Multimedia component 1 [file mmc1.docx]

Supplementary methods:

1. Activity assay of thioredoxins:

Thioredoxin activity assay was performed as described by Akif *et al* [1]. Insulin was used as a substrate to check reduction activity of TrxB, TrxB C33S, TrxC, TrxC C40S, NrdH, NrdH C14S. Briefly, the reaction mixture contains 100 mM sodium phosphate buffer pH 6.5, 2 mM EDTA, 1 mM DTT, 135 µM insulin. The final protein concentration used for the assay was 8 µM. Reaction was started by addition of 1 mM DTT at 25°C and precipitation of insulin was measured at 650 nm. Reaction without protein was selected as controls for the assay.

2. Far-UV Circular dichroism:

Far-UV Circular dichroism (wavelength range 190-260nm) experiment, was performed for TrxB, TrxB C33S, TrxC, TrxC C40S, NrdH, and NrdH C14S to show that the C to S mutations do not affect its folding. Jasco J-1500 instrument was used to perform the CD experiment. Protein concentration was 0.1 mg/ml in 10 mM Tris-Cl pH 8. Proteins The path length was 0.1 cm. CD data was collected for 250-190 nm wavelength at room temperature. Spectra was obtained in milli degree plotted against wavelength. Further, calculations for secondary structure prediction were done using BESTSEL online available software.
